# XSMILES: interactive visualization for molecules, SMILES and XAI attribution scores

**DOI:** 10.1186/s13321-022-00673-w

**Published:** 2023-01-06

**Authors:** Henry Heberle, Linlin Zhao, Sebastian Schmidt, Thomas Wolf, Julian Heinrich

**Affiliations:** 1 Division Crop Science, Bayer AG, 40789 Monheim am Rhein, Germany; 2 Division Crop Science, Bayer AG, 65926 Frankfurt, Germany

**Keywords:** SMILES, Molecule, Explainable artificial intelligence, Visualization, Artificial intelligence, Contribution, Attribution

## Abstract

**Background:**

Explainable artificial intelligence (XAI) methods have shown increasing applicability in chemistry. In this context, visualization techniques can highlight regions of a molecule to reveal their influence over a predicted property. For this purpose, some XAI techniques calculate attribution scores associated with tokens of SMILES strings or with atoms of a molecule. While an association of a score with an atom can be directly visually represented on a molecule diagram, scores computed for SMILES non-atom tokens cannot. For instance, a substring *[N+]* contains 3 non-atom tokens, i.e., [, $+$, and ], and their attributions, depending on the model, are not necessarily revealing an influence of the nitrogen atom over the predicted property; for that reason, it is not possible to represent the scores on a molecule diagram. Moreover, SMILES’s notation is complex, foregrounding the need for techniques to facilitate the analysis of explanations associated with their tokens.

**Results:**

We propose XSMILES, an interactive visualization technique, to explore explainable artificial intelligence attributions scores and support the interpretation of SMILES. Users can input any type of score attributed to atom and non-atom tokens and visualize them on top of a 2D molecule diagram coordinated with a bar chart that represents a SMILES string. We demonstrate how attributions calculated for SMILES strings can be evaluated and better interpreted through interactivity with two use cases.

**Conclusions:**

Data scientists can use XSMILES to understand their models’ behavior and compare multiple modeling approaches. The tool provides a set of parameters to adapt the visualization to users’ needs and it can be integrated into different platforms. We believe XSMILES can support data scientists to develop, improve, and communicate their models by making it easier to identify patterns and compare attributions through interactive exploratory visualization.

**Supplementary Information:**

The online version contains supplementary material available at 10.1186/s13321-022-00673-w.

## Introduction

Artificial Intelligence (AI) models have varied applications in chemistry, such as molecular property prediction [1, 2]. Chemists can use models to predict characteristics of small molecules in silico experiments, for instance, to identify compounds expected to be soluble or to have a certain bioactivity. Not only the analyses performed with predictions help scientists to identify potential candidates for further experiments, it can accelerate the discovery of new products and reduce costs with data-driven prioritization of candidate targets for further experimentation—e.g., in-vivo studies.

In typical silico screening processes, computational chemists and data scientists analyze substructures and identify patterns that may be influencing the predicted properties—e.g., bioactivity, solubility, or reactivity. Explainable artificial intelligence (XAI) techniques have been used to understand the behavior of models by calculating scores of influence of substructures over the predicted property [3–5]—here defined as *attribution scores* or simply *attributions*, also found in the literature as *attention*, *heatmap*, *coloring*, and *sensitivity scores* [6–9], depending on the methods and context. Visualization techniques can make the attribution scores more understandable, for instance, by coloring atoms of a 2-dimensional molecule diagram (i.e., structural formula diagram).

Although visualization is essential to interpret XAI attributions, few tools are available to help data scientists, computational chemists, and chemists to screen sets of molecules while analyzing their XAI attributions [10]. RDKit allows to highlight regions of a molecule diagram quantitatively and qualitatively, for example, by expressing different values of atom-attributions and by highlighting regions only expressing whether or not they are important, respectively. Both approaches are useful in numerous situations, for example, quantitative analysis may be preferred by some AI and XAI developers because of the detailed information, a qualitative approach may be an attractive option for some users, e.g., chemists and regulatory agents, who are more interested in overview and highlight of the crucial parts of a molecule.

A few authors have adapted or combined graphics to represent atom and non-atom attributions in a *static approach*—e.g., from non-atom tokens of a SMILES string, a machine-readable single-line string [11] that encodes a molecular structure. SMILES tokens are mostly characters representing atoms like C and N, and non-atom characters that describe the SMILES structure, like branches that are represented by parenthesis; additionally, some tokens can be formed by two characters, like Cl and Br. Karpov et al. used a bar chart in their Figure 8 [5] to represent atom contributions aligned with a SMILES representation, side-by-side with a molecule diagram. They used colors in both cases to indicate if the atoms stand for mutagenic alerts or against it. Lambard and Gracheva used a bar chart, heatmaps and a molecule diagram side-by-side in their Figure 6 [12] to represent the importance of substructures concerning atom and non-atom tokens from a SMILES string. The authors of both mentioned articles had to create the visualizations separately and join them into the mentioned figures. While the task of analyzing non-atom attributions can be achieved by a combination of graphics, this approach is time-demanding and is usually not coordinated nor interactive. The process becomes impractical and difficult for a larger set of molecules.

**Fig. 1:**
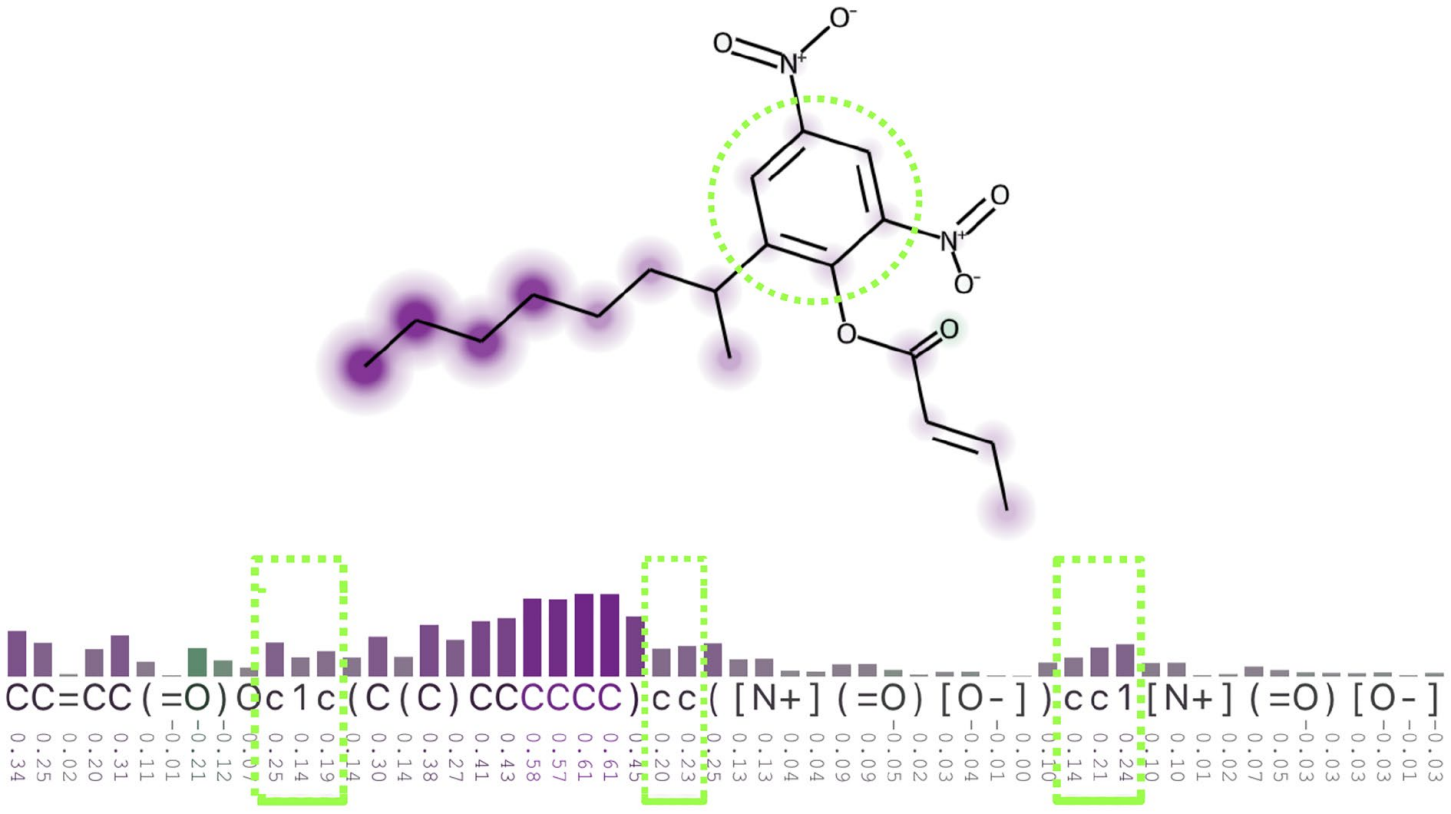
Visualization of a molecular structure and respective SMILES string. The dotted circle highlights the ring from the molecule, and the dotted rectangles highlight the tokens that represent the ring, exemplifying the complexity of the SMILES notation. The heatmap and bar chart represent attribution scores. The image was adapted from an XSMILES visualization

Another example of limitation of such a *static approach* is that, due to the complexity of SMILES’ syntax, strings are generally difficult to interpret and to identify which part of the molecular structure a set of chars is representing. Figure 1 illustrates how atoms that are close in a molecule (dotted circle) can be distant in a SMILES representation (dotted rectangles). Although the ring’s tokens are far in the string, they received similar attributions. If the supposed predictive model was trained at the SMILES token level, the visualization raises the hypothesis that the model may have learned patterns from the actual molecular structure.

To support the analysis of atom and non-atom attributions, we propose XSMILES (eXplainable SMILES), an interactive visualization technique to explore and compare atom and non-atom scores and support the interpretation of SMILES. XSMILES represents attributions on a 2D molecule diagram and a coordinated bar chart that represents a SMILES string. Its flexibility allows broad application, such as for representing magnitude or for highlighting parts of a molecule, and its interactivity makes SMILES strings easier to interpret. We implemented the technique in JavaScript and made it available as a plugin for JupyterLab, as a web-tool, a JavaScript package, and as a KNIME [13] component, making XSMILES an option in multiple frameworks. Moreover, it improves the analysis of multiple molecules, since it can replace the mentioned combination of static bar charts and molecule diagrams with interactive visualizations.

In the following sections, we explain how we designed the visualization and identified the main requirements that guided our project. Following those statements, we demonstrate the use of XSMILES to analyze output from a variety of XAI methods through two use cases.

## The XSMILES technique

While developing AI models and XAI techniques that have a SMILES string as an input, we identified a gap: there is no interactive visualization technique to support tasks involving interpreting SMILES-based attributions. Moreover, analyzing only atom attributions—i.e., ignoring non-atom tokens—using heatmaps on molecule diagrams were not enough to interpret the behavior of the SMILES-based models that we have been studying—this is exemplified in *Use case 1*. Based on this experience, we identified a list of main requirements (R) to develop a visualization technique that can help data scientists and computational chemists to analyze such types of models:

- *R SMILES*: Representation of atom and non-atom attributions. *Why* XAI methods and models based on SMILES strings require analyses that explore information associated with all tokens from sets of SMILES strings. *What* XAI methods output attributions mapped to a sequence of tokens. *How* Atom and non-atom attributions are visualized through bar charts.
- *R Molecule*: Representation of the molecular structure. *Why* Molecule diagrams are much easier to understand than SMILES strings. The goal of the analysis is not only to understand attributions based on a sequence of tokens alone, but also to identify patterns between sub-strings of the sequence and the chemical structure. *What* SMILES tokens translate to atoms or substructures of a molecule. *How* An interactive molecule diagram is coordinated with the bar chart, revealing what each token represents when users interact with them.
- *R Flexibility*: Interactivity and customization of the visualizations. *Why* AI models and XAI techniques output attributions of different nature. When developing them, the magnitude, the sign, and the sets of attributions that meet certain criteria need to be interpreted under different perspectives, requiring a flexible visualization tool. *What* Attribution scores need to be mapped to different visual representations to enable the analysis. *How* A set of parameters can be adjusted by users, e.g., color palettes and how the colors on the bar chart and molecule diagram are mapped to attributions.

**Fig. 2:**
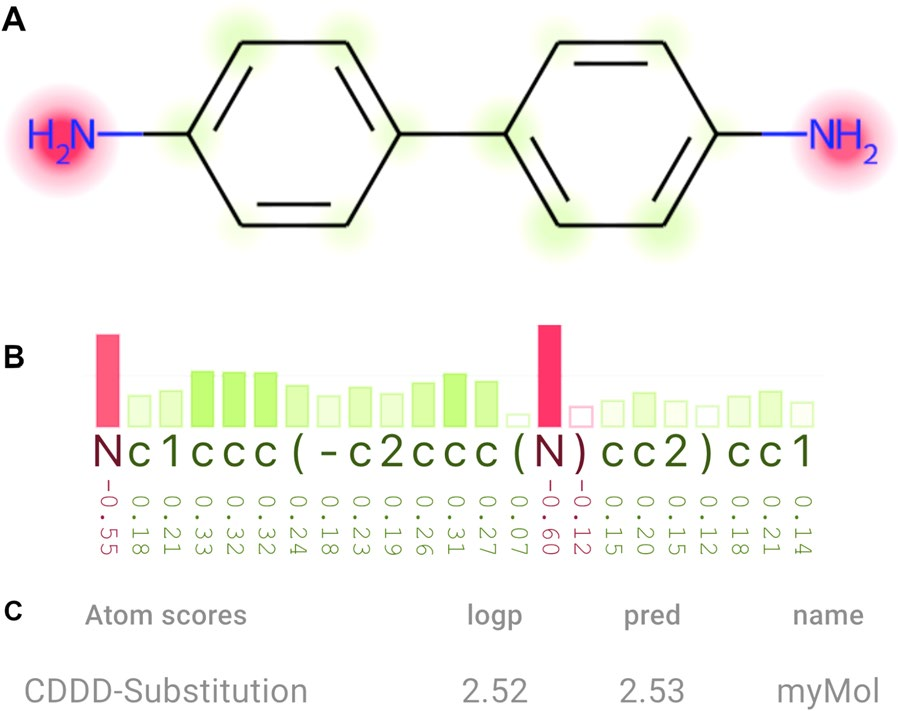
XSMILES has three main components. **A** A *molecule diagram* is displayed over a heatmap that represents atom attributions. **B** A *bar chart* represents a SMILES string and its associated attributions. **C** *Attributes* can be defined by the user in a tabular format

The requirements defined above summarize why and how we designed the XSMILES technique. In the following paragraphs, we explain each visualization component (see Fig. 2), color-related features, and interactivity.

*Attributes* Because each molecule is associated with certain properties and predictions, and can have its attributions represented by different color scales, we defined a table that is displayed under the Bar chart. The table can indicate information set by the user and is optional, i.e., can also be hidden.

*Bar chart* We designed a bar chart combined with colors to represent attributions from each SMILES token due to the improved interpretation of magnitude in contrast to using colors only in a heatmap. A diverging palette and the negative sign (−) or its absence under the bars inform the user if the attribution contradicts or supports the predicted property, respectively. The size of bars represents the magnitude of the attribution. If magnitude is not relevant, users can change the *colormap domain* to set all bars to the same height (see paragraph *Colormap domain*). The input for the XSMILES can be a set of attributions for all tokens or a smaller set with attributions only for atom-tokens. In the latter case, we attribute 0.0 to non-atom tokens. By default, two horizontal lines represent where the min and max values stand in the height direction of the bar chart.

*Molecule diagram* We chose RDKit to draw the molecules based on its increasing popularity. We used heatmaps placed on top of the molecule diagram to represent the attributions. Colors of the heatmaps are aligned with colors of the bar chart. Users can choose if atoms and bonds from the molecule diagram will be colored according to the atom-colors defined by RDKit, or if atoms are colored in black.

*Color palettes and sign direction* XSMILES has a predefined set of diverging color palettes that are intended to be colorblind friendly . Additionally, users can define custom diverging palettes. We created the default palettes based on Color Brewer [14] color schemes, aiming for colors that could differentiate the sign direction of attributions. All palettes go through an interpolation and lightness correction process. Signs represented by atom labels on the molecule diagram (as seen at the top-left of Fig. 4) and under the bar chart, as well as interactivity, help users to identify the sign direction of a certain attribution when color difference is not perceptible by the user. Throughout the article, we used different palettes in the figures to exemplify them.

**Fig. 3:**
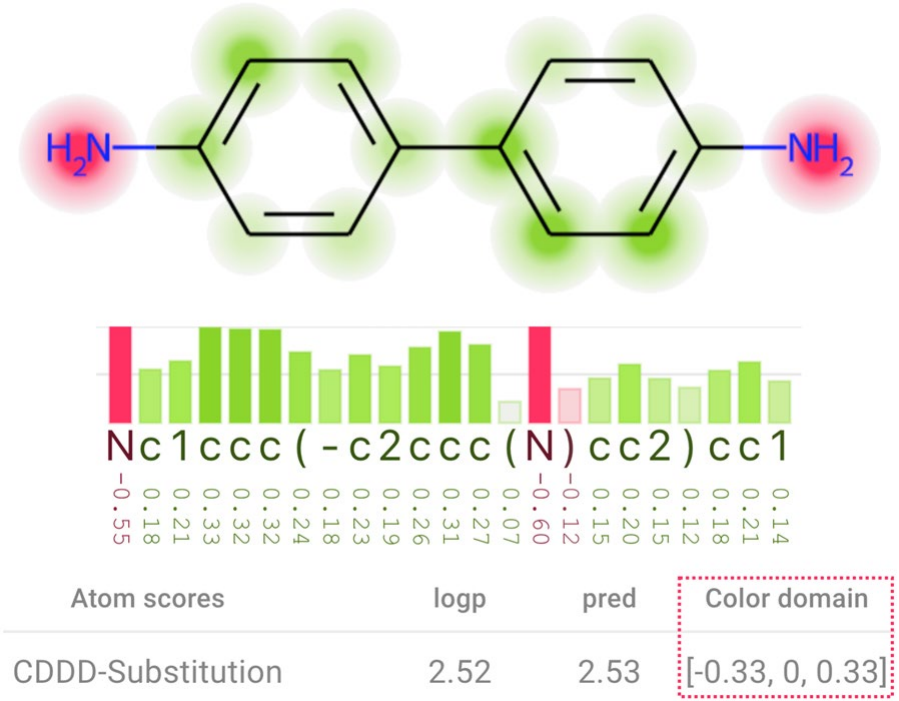
The color domain is manually set to range from − 0.33 to 0.33 instead of − 0.6 and 0.6 as shown in Fig. 2, which uses the maximum absolute value among all attributions of this molecule (0.6) to define the color domain. Here, values above or equal to 0.33, or below or equal to − 0.33, are represented by bars with maximum size and strongest colors, and by the strongest colors and largest areas in the heatmap. In comparison with Fig. 2, this visualization emphasizes more the attributions that are closer to zero

*Colormap domain* Users can define the colormap domain (attributions’ domain) and the range (color range) so that any value smaller than the minimum or greater than the maximum attribution is considered as minimum or maximum, respectively. This is a flexible feature that allows users to highlight regions with attributions above or below a certain value with the strongest colors, as demonstrated in Fig. 3.

**Fig. 4:**
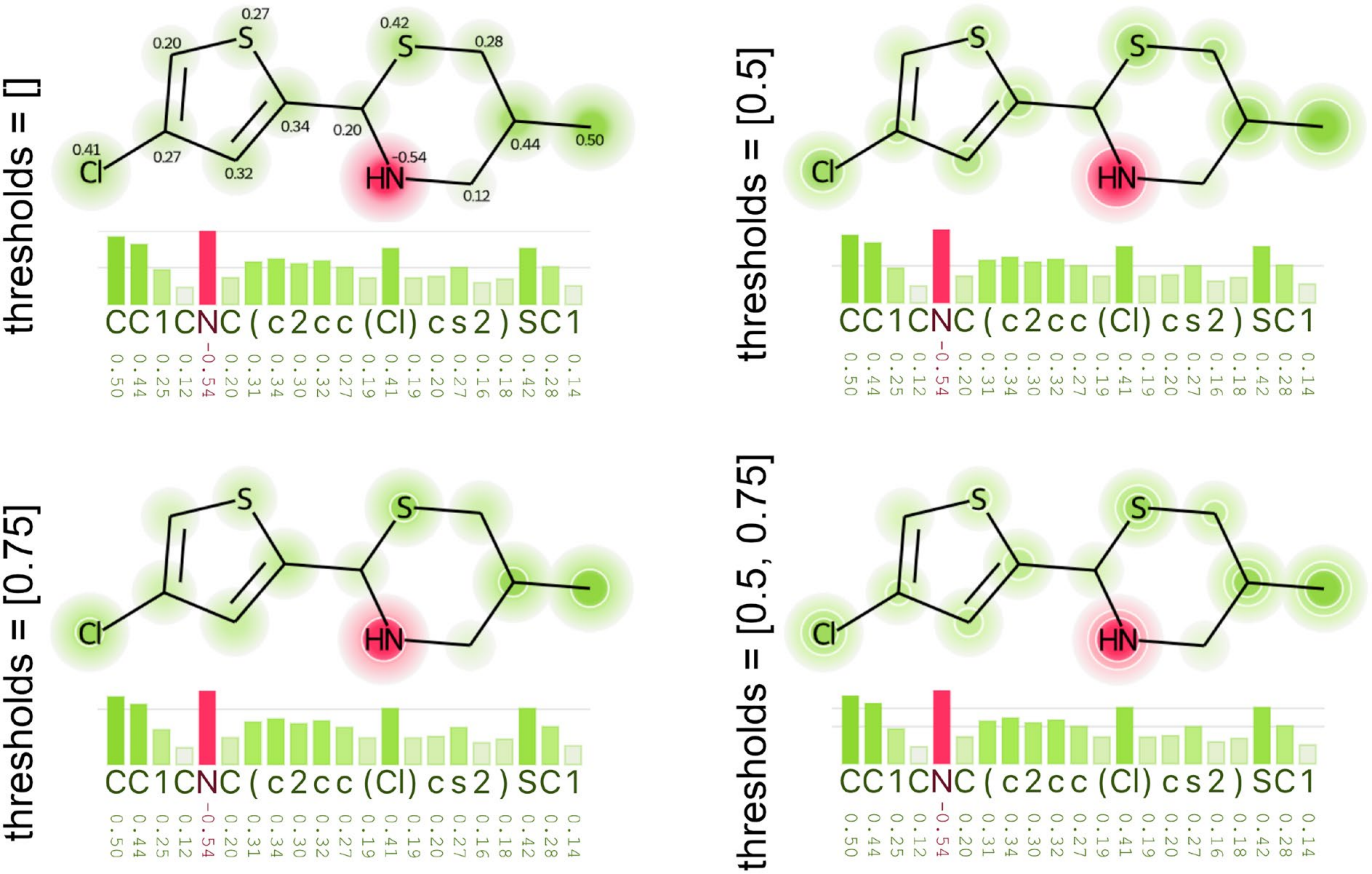
Thresholds help identifying atoms and tokens that have the absolute value of their attributions above certain values. Here we see four examples: the top-left one has no thresholds ([]) and indicate attributions with labels on the molecule diagram, and the three others have thresholds lists equal to [0.5], [0.75], and [0.5, 0.75]

*Threshold highlight and labels* Because comparing magnitude through an overview heatmap can be difficult, we implemented the possibility to set thresholds: values between 0 and 1 that are used to highlight atoms on the molecule diagram and create horizontal lines on the bar chart. It highlights attributions that have an absolute value above a percentage of the *colormap domain*’s maximum value. For example, with a $[-0.5, 0, 0.5]$ *color domain*, a threshold of 0.5 would highlight atoms with attributions greater or equal to 0.25 and lower or equal to $-0.25$. Figure 4 illustrates the visual effect of not using threshold ([]) and of using [0.5], [0.75], and [0.5, 0.75] as highlight thresholds. The color of the heatmap becomes stronger, and atoms that match the criteria are circled by an optional white stroke. The greater the circle, the farther the attribution from the threshold. Horizontal lines are drawn according to the defined thresholds. If no threshold is defined, it is drawn at values 0.5 and 1.0. Atom attributions can also be displayed as labels on the molecule diagram, close to each atom, as shown in the diagram with no thresholds ([]) in Fig. 4. The motivation behind the threshold highlighting was our interest in identifying medium and large attributions; defining what is large will depend on the XAI method and model.

**Fig. 5:**
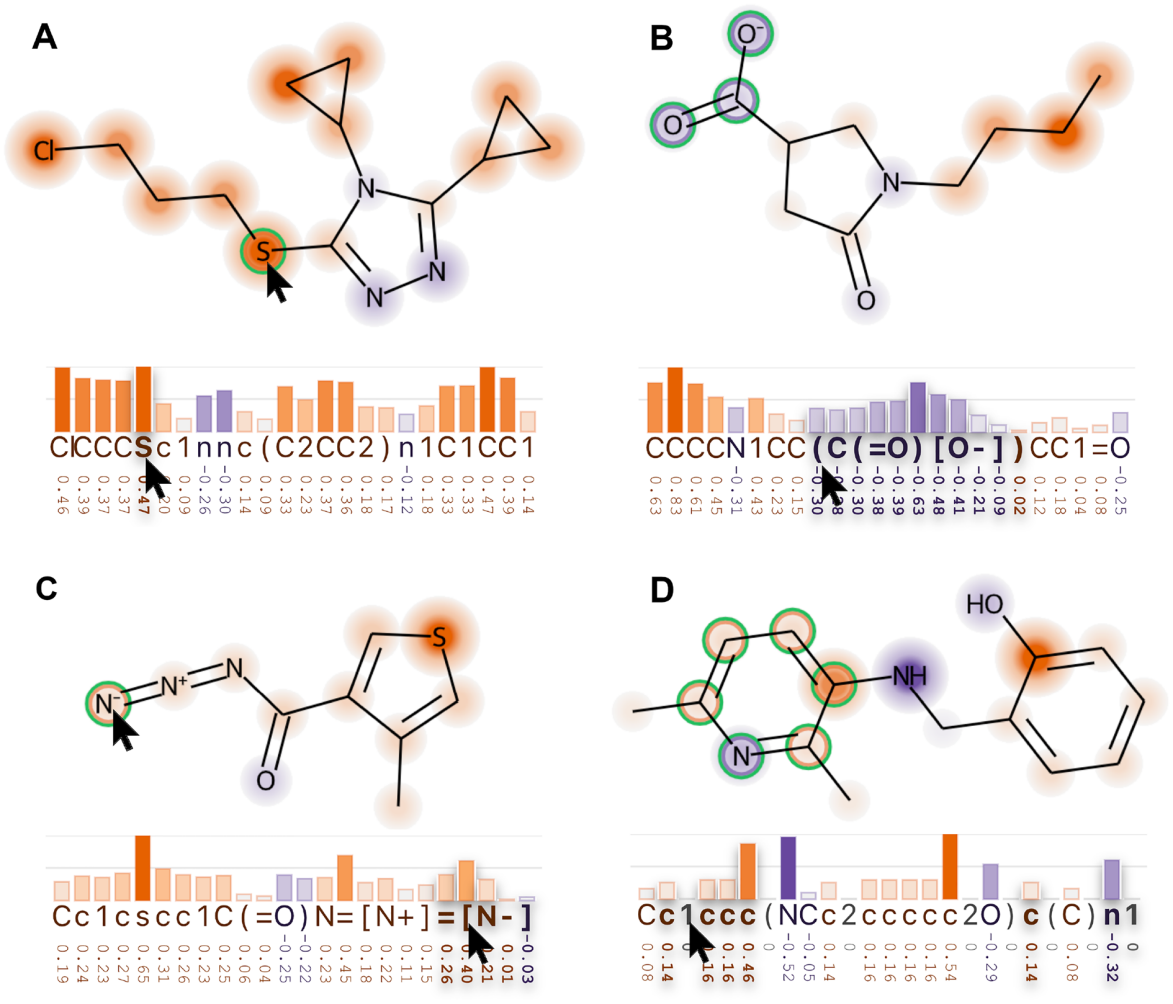
Four types of hover interaction. **A** The respective token is highlighted when you mouse over an atom, and vice-versa. **B** A branch is highlighted on the molecule and on the string when you mouse over a parenthesis character. **C** A group defined by square brackets is highlighted when you mouse over its tokens or its atom. **D** It highlights the ring on the molecule and on the string when you mouse over the numbers that encode that ring. When highlighted, the tool modifies the molecule representation to express the signal direction of the attribution clearer by drawing a circle around the atom with the signal direction’s color and another with the highlight color

*Interactivity* Users can hover atoms on the diagram to highlight the respective SMILES token, and hover the tokens to highlight substructures on the diagram. The highlighted tokens become bolder and the atoms on the molecule are circled with two colors: (1) the *signal color* that represents the positive or the negative ranges of attributions and (2) the *highlight color*, defined to contrast with the two signal directions’ colors. Users can highlight the following substructures on the diagram:

- *atom*: hover an atom token to highlight the atom (Fig. 5A);
- *ring*: hover a number (definition of ring openings and closings) to highlight the ring (Fig. 5B);
- *group*: hover the square brackets or any token between them to highlight the group (Fig. 5C);
- *branch*: hover a parenthesis to highlight a SMILES branch (Fig. 5D).

## Implementation

XSMILES is available as a JavaScript library and integrated into other extensions. Users can use it in JupyterLab notebooks and in KNIME [13] pipelines, or through the demonstration website, where they upload a JSON file with molecules and attributions. Web developers can integrate XSMILES into other web-based systems using the JavaScript package.

Its version 0.5.7 uses RDKit MinimalLib 2022.03.1 [15, 16] to draw molecules and Heatmap.js 2.0.5 [17] to draw the heatmaps. Both heatmaps and molecule diagrams are independent web canvas layers and can be replaced with new variants by web developers. Two heatmaps are generated per molecule diagram, one for positive and one for negative attributions. Highlights under the molecule are built using canvas elements, and bar charts are created with SVG elements. We used React [18] as the main framework to connect everything into the interactive visualization technique. Other required libraries and installation details are described in XSMILES’ source-code repository.

*Input format* XSMILES can be used to represent a molecule with or without atom or token scores. The order of tokens in the SMILES string defines the order of the scores vector. We use the same order used by RDKit, i.e., the first atom in the SMILES string will be the first node of the graph that RDKit uses to draw the structure. The scores vectors can be of two sizes: number of atoms and number of SMILES tokens, as exemplified in Use Case 2. In the first case, the score vector represent only atom-scores while in the latter, special tokens, like (, ], and +, also receive scores. The heatmap only considers atom-scores and does not take into account interactivity between atoms or functional groups. We define the input format in the GitHub repository with examples. The tool was tested with RDKit canonical SMILES.

*Atoms’ coordinates* The current version uses RDKit MinimalLib to generate two equal diagrams for each molecule: one in SVG format and one as a canvas element. XSMILES parses multiple SVG elements to derive the coordinates (x, y) of each atom in the diagram. We use this information to draw the heatmap and track the mouse pointer to identify when it is over an atom.

*Response time* The website version demonstrated to have instant response time in terms of interactivity when displaying over 100 XSMILES diagrams with molecule diagram, bar chart, and attribute table in our tests. However, loading time, i.e., processing JSON and initial rendering time, was not instant when loading over 100 molecules; a delay happens as well if parameters of the visualization are changed due to re-rendering time. Loading time with 42 diagrams (e.g., 14 molecules and 3 XAI methods), was close to instant in our test (macOS 11, Chrome 103 browser, Intel Core i7-9750H 2.6 GHz processor, 32 GB RAM, AMD Radeon Pro 5300M 4 GB graphic card). Given that most common computers don’t have a powerful configuration, we would recommend using around 20 molecules. Loading time for JupyterLab is different, since in that case, we may explore smaller sets of molecules loaded in different times.

*Availability* The tool is open-source and available at https://github.com/Bayer-Group/xsmiles. The plugin for JupyterLab, for KNIME, demonstration website and datasets availability is described in details in section Availability of data and materials.

## Use cases

### Use case 1—analyzing attributions and developing a bioconcentration factor model

The Bioconcentration Factor (BCF) quantifies a chemical’s potential to accumulate in living organisms, most frequently fish. As such, it is an important characteristic in the environmental risk assessment of chemicals. Zhao et al. [9], including authors of the XSMILES, created a model called xBCF that can predict BCF and provides attributions for SMILES strings.

In summary, xBCF is a deep learning model based on CDDD [19] molecular representations that use SMILES strings as input. The XAI method first substitutes the token of interest to any token in the vocabulary set of the CDDD model. Then the difference between the prediction from the original SMILES and the average prediction from all substituted SMILES is regarded as the attribution of the token of interest: the sensitivity score. A positive attribution indicates that the predicted BCF value is expected to drop when that token was substituted with any other token in the vocabulary.

The xBCF model was trained on public BCF data and internal logD data so that it can predict both logBCF and logD simultaneously. LogD represents the distribution coefficient of a chemical between octanol and water, where octanol is often seen as a proxy for organic tissue. This multitasking nature of xBCF was driven by the high correlation between logBCF and logD. Therefore, when the XAI method is applied on the xBCF model, one can obtain explanations for both logBCF and logD predictions which enable chemists to gain insights into the predictions and the model.

During the xBCF development, patterns of SMILES non-atom and atom tokens were analyzed for many molecules. Due to its dependency on the CDDD molecular representations encoded from SMILES strings, non-atom tokens played a key role in the translational autoencoder and the downstream predictive models for BCF and logD.

XSMILES was developed iteratively with the development of xBCF and was of great importance for the authors to analyze results during and after the development process. The model is now deployed *in house*, and XSMILES is used to display results to end users through interactive visualization. The XAI Substitution method is open-source and publicly available (see section Availability of data and materials). Detailed explanations about both model and XAI method are found in the original article [9].

**Fig. 6:**
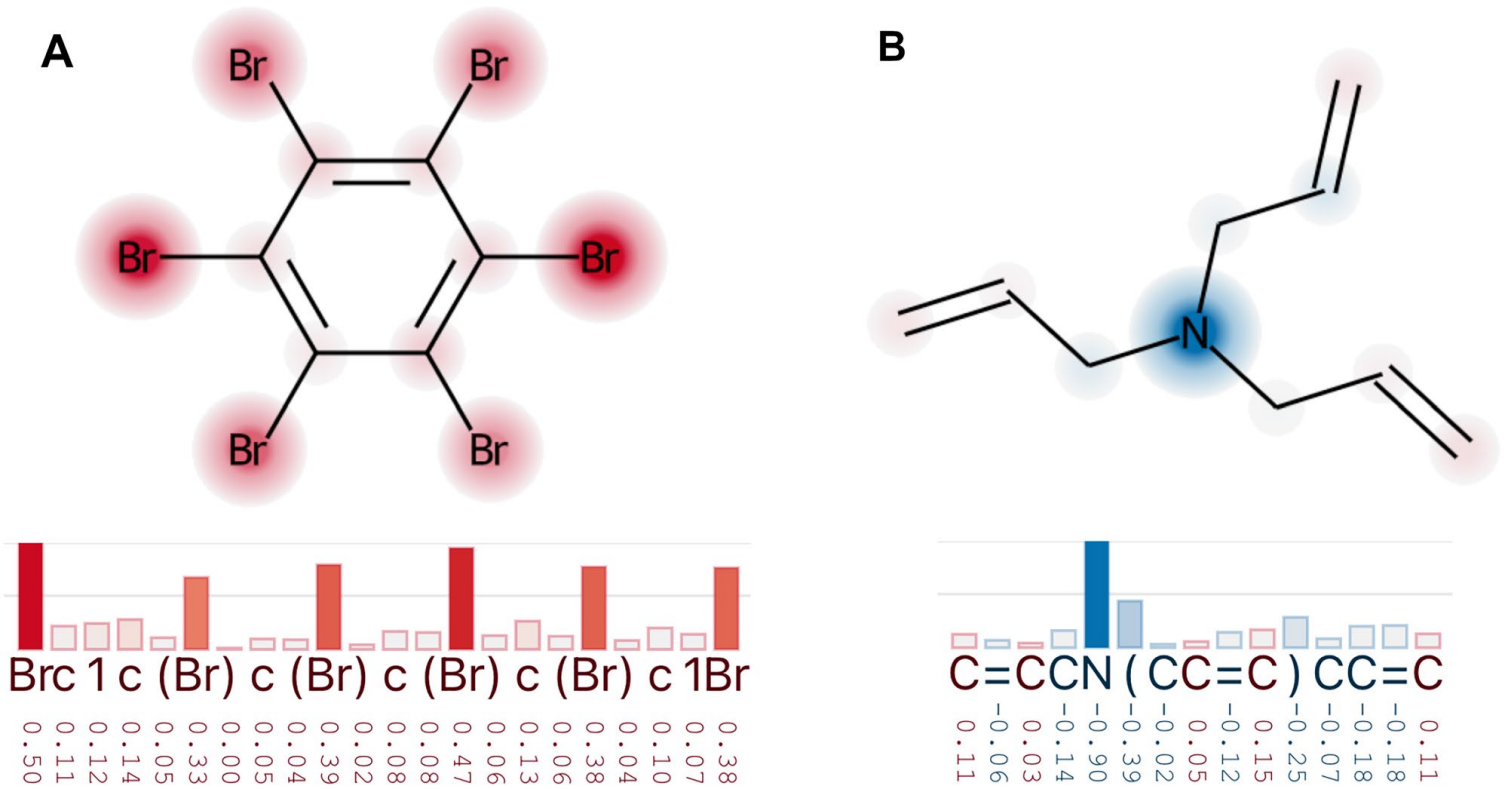
**A** All bromine atoms in hexabromobenzene were assigned similarly high logD sensitivity scores. **B** In triallylamine the three symmetry-equivalent allyl groups show similar low logBCF sensitivity scores while the central nitrogen has a large negative score

Zhao et al. [9] extensively used XSMILES to analyze how their model and XAI methods work. In Fig. 6 we reproduced examples illustrating xBCF model is able to recognize symmetry-equivalent functional groups and attributes similar sensitivity scores to equivalent atoms. Despite almost perfect symmetric attributions, it’s important to note that this was not always the case and regardless of results, XSMILES played a key role in the process of quickly screening molecules, identifying patterns and creating hypotheses.

**Fig. 7:**
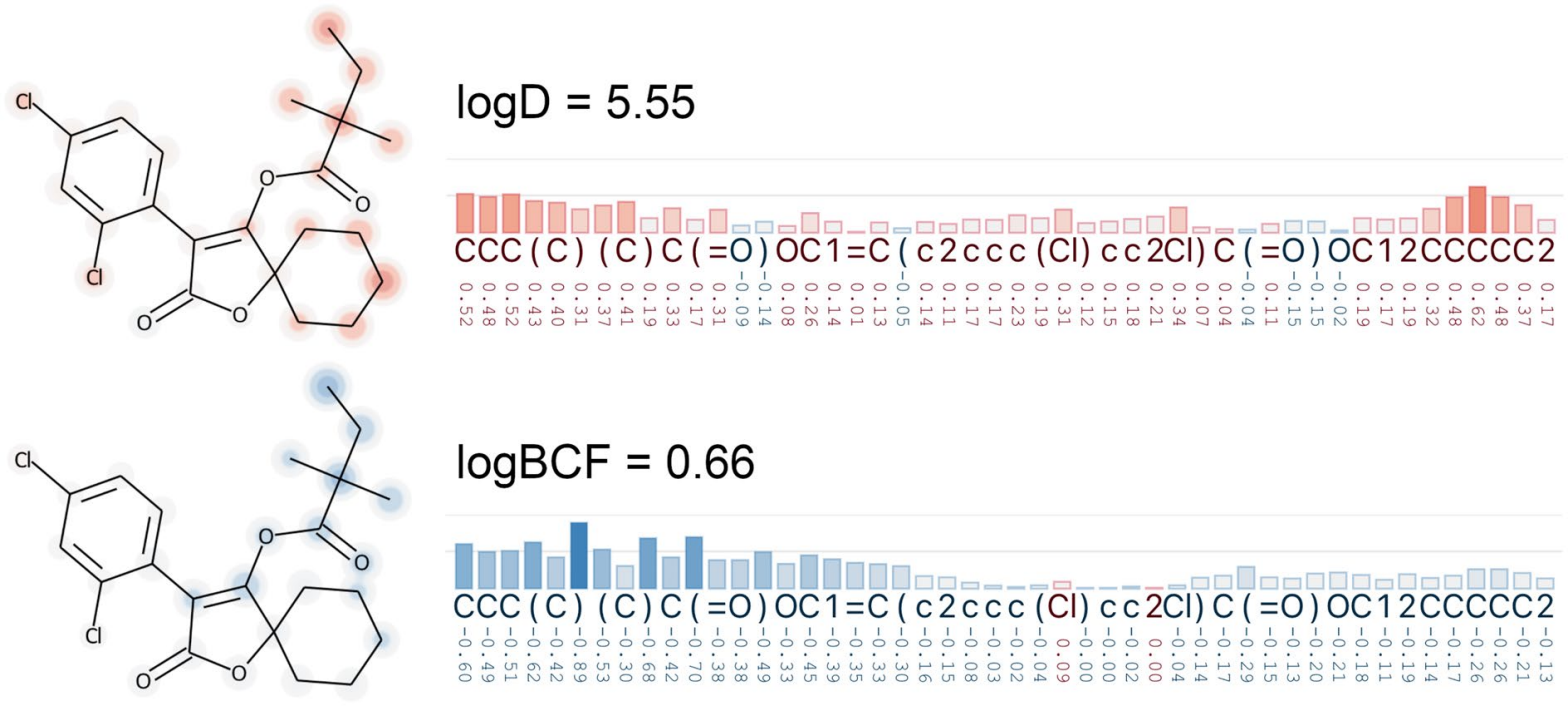
Spirodiclofen is a molecule with low logBCF (0.66) and high logD (5.5) predicted values. The sensitivity scores attributed to the SMILES tokens based on both logBCF and logD are similar, but have mostly the opposite sign direction—positive and negative, respectively. Both color-domains in this figure range from − 1 to 1

Another activity described by the authors is the comparison of logD and logBCF. In Fig. 7 we illustrate one of their examples with high logD (5.5) and low logBCF (0.66) predicted values: spirodiclofen, a molecule known to be readily metabolized. We see that the sensitivity scores for important parts of the molecule have different signs, which means that logD cannot explain the low BCF value.

Another aspect that helped the development of the xBCF was the fact that the authors could output a JSON file and quickly share with colleagues, and visualize results, without setting up any coding environment. The file with the molecules of this use case is available at our git repository and can be visualized with the XSMILES demonstration website.

Having the possibility of using XSMILES from within JupyterLab notebooks also helped them to quickly test and re-render visualizations based on new parameters defined to train the models, to develop the Substitution method or to adapt the visualization to better highlight patterns from the attributions.

In this use case, we described how XSMILES assisted the development of the xBCF model and is being used by end-users. The importance of the XSMILES was highlighted through examples of analysis that helped the xBCF’s authors to develop the model and the Substitution XAI method —both based on SMILES strings.

### Use case 2—analyzing logP attributions against Crippen logP atomic contributions

Rasmussen et al. [8] studied the original and transformed logP Crippen contributions as a potential ground truth to attributions calculated with the “atom attribution from fingerprints”-method developed by Riniker and Landrum [20] (in this text referenced as **R &L**). They compared the overlap of heatmaps between this attribution method and the original (atom-based) and adapted (fragment-based) logP atomic contributions. Throughout their analysis, they visually compared contributions with attributions, highlighting molecules with high and low heatmap overlap.

Here, we explore this idea of using logP contributions and comparing them with attributions, but with three different XAI methods. We visually compare the original logP atomic contributions calculated with RDKit against the R &L attributions and attributions from two additional approaches: one based on the *SMILES strings token-substitution method* [9] described in *Use case 1* and one based on Morgan fingerprints [21] and SHAP [10, 22, 23] values. A JupyterLab notebook with all methods is available (see section Availability of data and materials).

To calculate attributions, we combined the three attribution methods to two different CatBoost [24] (*catboost 1.0.5, iterations=10000, depth=6*) regressors, with a total of three different setups:

- *CDDD-Substitution*) a model trained with CDDD [19] molecular representations with attributions calculated using the Substitution method [9],
- *Morgan-SHAP*) a model trained with Morgan fingerprint bits (radius 1) with attributions calculated through the SHAP method [22], and
- *Morgan-R &L*) the same fingerprint-based model as the latter, but with attributions calculated using Riniker and Landrum’s method [20].

Overall, the predictions from the two Catboost regression models resulted in good coefficient of determination (above 0.9) and root mean squared error (below 0.19). More details about the models that we tested and performance are described in Additional file 1.

We analyzed the attributions from the CDDD-Substitution, Morgan-SHAP, and Morgan-R &L methods. Note that there are significant differences among the compared methods regarding XAI techniques (R &L, SHAP, Substitution), molecular representation (Morgan, CDDD), and predictive performance. This use case shows how we can explore their calculated attributions with XSMILES to create hypothesis and inspire thoughts.

To visualize dozens of molecules, we generated JSON files describing their calculated attributions. These datasets were then visualized using the demonstration website available at the project’s main repository. The website provides the user the capability of quickly visualizing sets of molecules and their attributions, and of changing XSMILES’ parameters, such as color palette, color domain, and thresholds. Here we focus on one molecule that we found to be quantitatively and qualitatively very interesting.

**Fig. 8:**
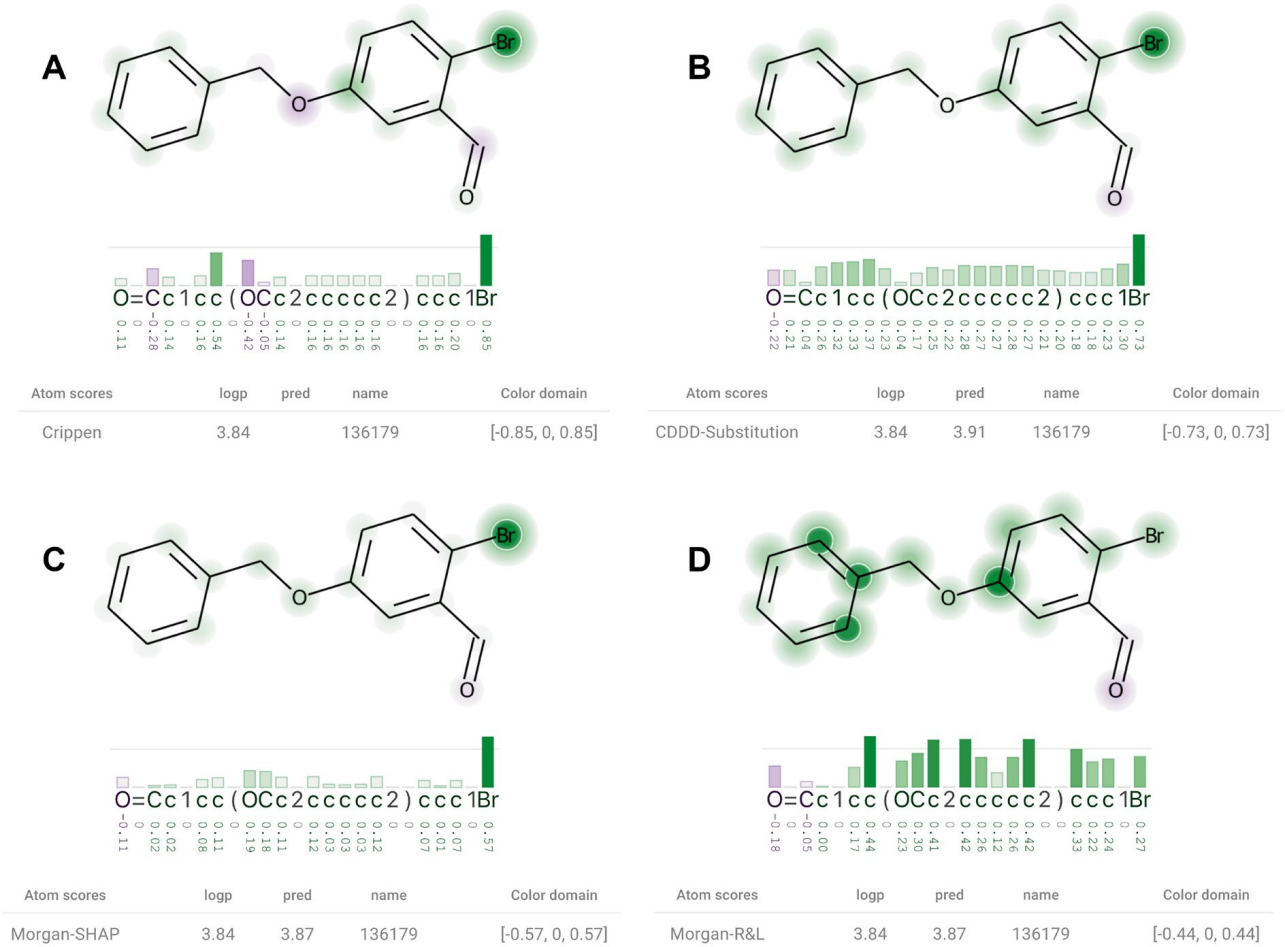
**A** The Crippen contributions to logP. **B** Attributions from CDDD-Substitution are similar to the ones found in A regarding their sign, but the most influential oxygen is not highlighted as much as in A. Attributions from Morgan-SHAP (**C**) and Morgan-R &L (**D**) are almost identical to A (relative to their own maximum absolute value)

Figure 8 shows diagrams where the *color domain* was defined for each molecule based on their maximum absolute score. With a threshold of 0.75, the diagram highlights with white circles and darker colors the most influential atoms, and displays a horizontal line to help to identify tokens that overpass the threshold. The logP contribution (A) of the bromine (last token from the SMILES string) is highly positive. While CDDD-Substitution (B) and Morgan-SHAP (C) identified the same bromine as the most influential atom, Morgan-R &L (D) attributed the highest values to carbons. An explanation for this difference could be the molecular representation, which is not atom-based but fragment-based, as made clear by Rasmussen et al. [8]. However, Morgan-SHAP uses the same molecular representation and spotlighted the bromine similarly to the contributions.

Although the CDDD-Substitution (B) highlighted the bromine atom in Fig. 8, it attributed higher values to the carbons than the ones we find in the contributions vector (A). Moreover, it considers the prediction to be as sensitive to a substitution of non-atom tokens as to a substitution of carbons, in general. This highlights that the CDDD model utilizes the non-atom tokens to correctly represent the molecular structure, as opposed to reading only a linear chain of atoms.

**Fig. 9:**
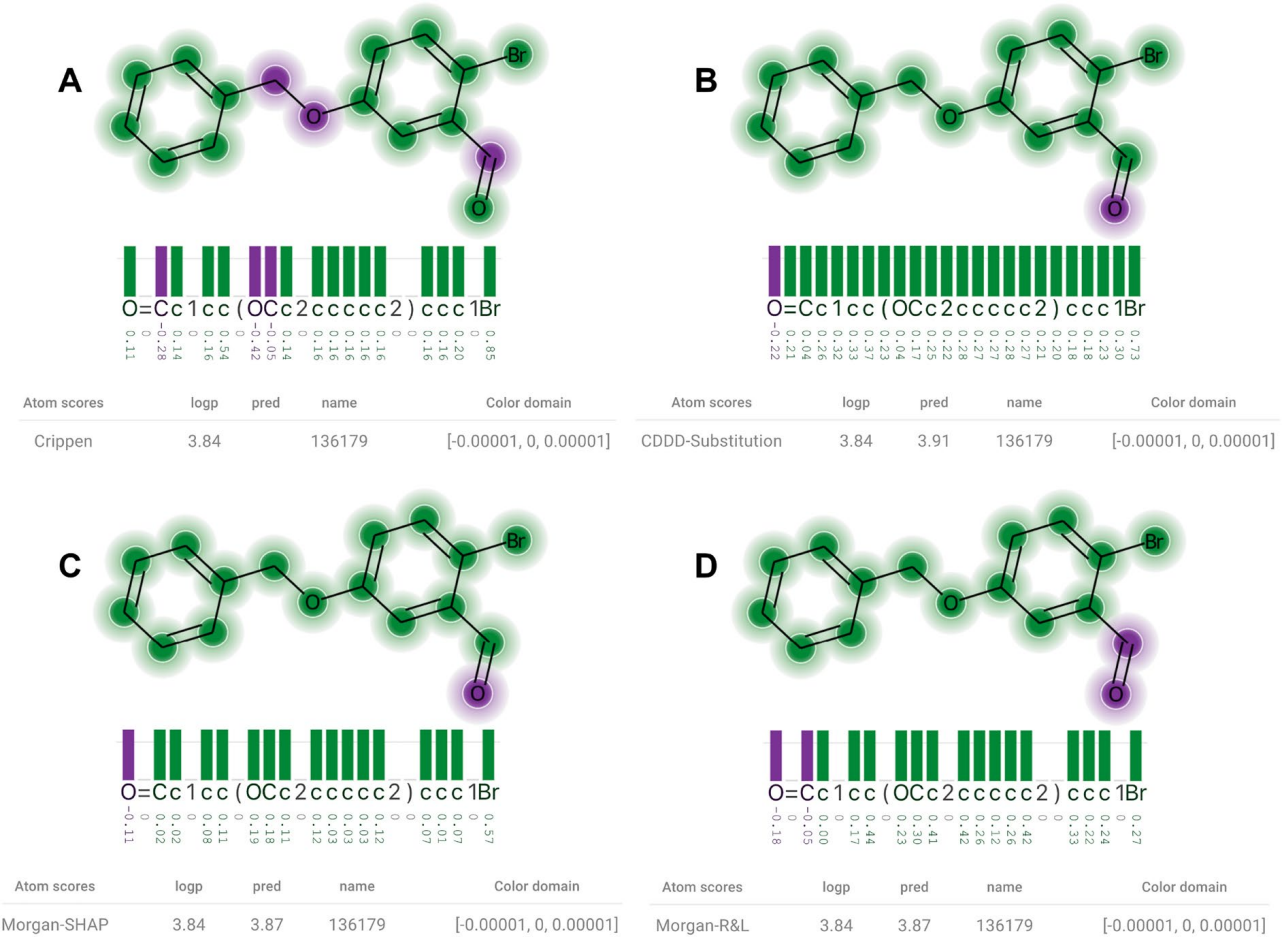
Crippen Contributions (logP) and attributions extracted from different models are visualized with a table indicating the method, ground truth (logp), and prediction (pred). A small value was used to define the *color domain* so that the visualization express only the sign of the scores. **A** The Crippen contributions to logP. **B** Attributions from Substitution-CDDD. **C** Attributions from Morgan-SHAP. **D** Attributions from Morgan-R &L

Although quantitatively the same bromine was highlighted by the contributions (A), CDDD-Substitution (B) and Morgan-SHAP (C) in Fig. 8, the story changes if we analyze them qualitatively through the direction of attributions’ sings. In Fig. 9 we see that the contributions for the two oxygen atoms are different: positive for the first oxygen token in the string, and negative for the second. All the three methods (B, C, D) attributed the opposite direction to both oxygen atoms. As of additional information, all atoms have positive contribution in the FPA contributions, which also disagrees with the three methods. To ignore the magnitude in this example, we set the *color domain* to be equal to a tiny value, i.e., between − 0.00001 and 0.00001. With this approach, all attributions are represented equally in terms of absolute values.

In this use case, we demonstrated how XSMILES can be used to compare attributions from different methods. We used methods based on atom-attributions only and one method based on SMILES-attributions. Although the models and molecule representations differ drastically, we found many cases in which attributions created by each method are relatively similar to the logP contributions. In other cases, attributions would agree among themselves and disagree with the contributions. The analysis gets complex and XSMILES has helped in the task of identifying patterns and facts that agree and disagree with our beliefs about the methods, models, and molecular representations.

## Final considerations

Data scientists can use XSMILES to understand their models’ behavior and compare multiple approaches. With our use cases, we demonstrated how attributions calculated for SMILES strings can be evaluated and better interpreted through interactivity. Furthermore, we exemplified how a side-by-side approach may be used to compare different models and explanations, and how a website where users can quickly analyze molecules without a coding environment is useful.

XSMILES can be used to visualize not only XAI attributions, but any set of scores associated with atom or non-atom tokens of a SMILES string—e.g., attributions derived from models that are based on a graph representation instead of a SMILES one. It is also a good technique to learn the SMILES notation and interpret SMILES strings. Moreover, it uses RDKit’s drawing standard, works within JupyterLab, and can be integrated into other web-based architectures.

Among the ideas for improvements and new applications of such technique are the interactive visualization of SELFIES [25] and InChI [26], and the implementation of better ways to represent the attributions. For example, we coded XSMILES in a way that the drawing of the molecular structures could be done by other drawers like SmilesDrawer [27]. A set of different types of heatmaps algorithms and highlights could also be implemented and offered to users. Finally, XSMILES is open-source, and we believe it is a great contribution for the community.

## Supplementary Information

**Additional file 1.** Additional information for Use case 2.

## Data Availability

XSMILES is open-source and available at https://github.com/Bayer-Group/xsmiles, in which we provide the core information, an example of how to integrate the tool with KNIME, the access to the demonstration website, to the source-code from the JupyterLab implementation, and data and notebooks used in the use cases. We provide a notebook with scripts demonstrating how we trained LogP models and calculated attributions using the molecules downloaded from [8]. The datasets used to train the xBCF model and the method to calculate sensitivity scores are part of the xBCF article [9], please consult the original material for more information.

## Abbreviations

AI: Artificial intelligence
BCF: Bioconcentration factor
CDDD: Continuous and data-driven descriptors
InChI: International chemical identifier
JSON: JavaScript object notation
SHAP: Shapley additive explanations
SMILES: Simplified molecular input line entry system
SELFIES: Self-referencing embedded strings
SVG: Scalable vector graphics
XAI: Explainable AI
xBCF: Explainable bioconcentration factor model
XSMILES: Explainable SMILES
