# Supplementary material for: XSMILES: interactive visualization for molecules, SMILES and XAI attribution scores
Source: J Cheminform. 2023 Jan 6;15:2. doi: 10.1186/s13321-022-00673-w (PMC9817292; doi:10.1186/s13321-022-00673-w)
Supplement: Supplementary file 1 — Additional file 1. Additional information for Use case 2. [file 13321_2022_673_MOESM1_ESM.pdf]

Additional file 1

XSMILES: interactive visualization for molecules, SMILES and XAI scores

Henry Heberle, Linlin Zhao, Sebastian Schmidt, Thomas Wolf and Julian Heinrich

January 3, 2023

Use case 2 – Analyzing logP attributions against Crippen logP atomic contributions

Using a dataset of 249,435 molecules [1], we separated 80% for training and 20% for test, randomly sampled. We trained CatBoost [2] (*catboost 1.0.5, iterations=10000, depth=6*) regressors based on CDDD [3] to use with the Substitution XAI method (**CDDD-Substitution**) and Morgan fingerprints(2048 bits, radius 1) to use with SHAP (**Morgan-SHAP**) and Riniker and Landrum (**Morgan-R&L**) attributions methods [4, 5, 6, 7]. The reason we utilize CDDD with the xBCF Substitution method is that the substitution process generates invalid SMILES strings and, therefore, it is not possible to calculate Morgan fingerprint for those. LogP contributions were calculated with *rdMolDescriptors._CalcCrippenContribs* function from RDKit. A JupyterLab notebook with all details is available at https://github.com/Bayer-Group/xsmiles-use-cases.

In the XSMILES main repository, we provide a JSON file with the molecules used in this use case. Additionally, the file contains versions of transformed Crippen logP contributions that Rasmussen M. H. et al. [1] used in their study, namely *normalized Crippen* and *fingerprint adapted* (FPA).

Since Rasmussen M. H. et al. [1] used a random forest in their article, we decided to analyze how their model would perform with our test set and trained additional random forest models (*scikit-learn 0.23.2, default parameters*) using the two different molecular representations (2048 radius 2 Morgan fingerprints and CDDD).

In our tests, Catboost models showed, in general, better performance over random forest models. Therefore, we used Catboost models to calculate attribution for this use case. Another decision made was to use radius 1 when calculating Morgan fingerprints. Models trained on Morgan fingerprints with radius 1 performed better than with radius 2.

We didn't extensively test models, and it is not in the scope of this article. Table 1 summarizes the tested models and which XAI method we considered for each of them. The two CatBoost models that we use in this use case are indicated with an asterisk (*).

Table 1: The performance of tested regressors is indicated by coefficient of determination (R2) and root mean squared error (RMSE). The XAI column indicates the methods that can be used with each type of regressor without changing the source-code. The table indicates in bold the best performances and with an asterisk (*) the models used in this use case. Although CatBoost with Morgan fingerprint counts performed better in comparison with Morgan bits, we chose the latter because of its code-compatibility with SHAP and R&L. All performance scores were calculated with the same test set with approximately 50,000 molecules. Our models were trained with approximately 200,000 other molecules. The third Random Forest model was trained by Rasmussen M. H. et al. [1] – our test set may have overlap with their training set.

**Regressor**	**Representation**	**R2**	**RMSE**	**XAI**	**Training size**
Random Forest	CDDD	**0.97**	**0.04**	Substitution	200,000
Random Forest	Morgan bits (radius 1)	0.86	0.27	SHAP, R&L	200,000
Random Forest [1]	Morgan bits (radius 2)	0.84	0.32	SHAP, R&L	**150,000**
CatBoost*	CDDD	**0.99**	**0.009**	Substitution	200,000
CatBoost*	Morgan bits (radius 1)	0.91	0.18	SHAP, R&L	200,000
CatBoost	Morgan bits (radius 2)	0.90	0.19	SHAP, R&L	200,000
CatBoost	Morgan counts (radius 1)	**0.99**	**0.02**	SHAP	200,000

References

[1] Rasmussen, M.H., Christensen, D.S., Jensen, J.H.: Do machines dream of atoms? a quantitative molecular benchmark for explainable AI heatmaps (2022). doi:10.26434/chemrxiv-2022-gnq3w. This content is a preprint and has not been peer-reviewed. **[cito:containsAssertionFrom]** **[cito:usesDataFrom]** **[cito:usesMethodIn]**

[2] Dorogush, A.V., Ershov, V., Gulin, A.: Catboost: gradient boosting with categorical features support. arXiv preprint arXiv:1810.11363 (2018). doi:10.48550/arXiv.1810.11363. This content is a preprint and has not been peer-reviewed. **[cito:usesMethodIn]**

[3] Winter, R., Montanari, F., Noé, F., Clevert, D.-A.: Learning continuous and data-driven molecular descriptors by translating equivalent chemical representations **10**(6), 1692–1701 (2019). doi:10.1039/c8sc04175j. **[cito:usesMethodIn]**

[4] Lundberg, S.M., Lee, S.-I.: A unified approach to interpreting model predictions. Advances in neural information processing systems **30** (2017). **[cito:obtainsBackgroundFrom]** **[cito:usesMethodIn]**

[5] Rodríguez-Pérez, R., Bajorath, J.: Interpretation of compound activity predictions from complex machine learning models using local approximations and shapley values. Journal of medicinal chemistry **63**(16), 8761–8777 (2019). doi:10.1021/acs.jmedchem.9b01101. **[cito:usesMethodIn]**

[6] Humer, C., Heberle, H., Montanari, F., Wolf, T., Huber, F., Henderson, R., Heinrich, J., Streit, M.: ChemInformatics model explorer (CIME): exploratory analysis of chemical model explanations **14**(1), 1–14 (2022). doi:10.1186/s13321-022-00600-z. **[cito:obtainsBackgroundFrom]** **[cito:AuthorSelfCitation]** **[cito:usesMethodIn]**

[7] Riniker, S., Landrum, G.A.: Similarity maps - a visualization strategy for molecular fingerprints and machine-learning methods. Journal of cheminformatics **5**(23), 1–7 (2013). doi:10.1186/1758-2946-5-43. **[cito:citesForInformation]** **[cito:usesMethodIn]**
